# Fetal Exposure of Rhesus Macaques to Bisphenol A Alters Cellular Development of the Conducting Airway by Changing Epithelial Secretory Product Expression

**DOI:** 10.1289/ehp.1206064

**Published:** 2013-06-11

**Authors:** Laura S. Van Winkle, Shannon R. Murphy, Miriam V. Boetticher, Catherine A. VandeVoort

**Affiliations:** 1 Department of Anatomy, Physiology and Cell Biology, School of Veterinary Medicine,; 2 Center for Health and the Environment,; 3 Department of Obstetrics and Gynecology, School of Medicine, and; 4 California National Primate Research Center, University of California, Davis, Davis, California, USA

**Keywords:** CC10, CC16, lung development, *Macaca mulatta*, mucin, prenatal, SCGB1A1

## Abstract

Background: Bisphenol A (BPA) exposure early in life results in organizational changes in reproductive organs, but the effect of BPA on conducting airway cellular maturation has not been studied. Late gestation is characterized by active differentiation of secretory cells in the lung epithelium.

Objective: We evaluated the hypothesis that BPA exposure disrupts epithelial secretory cell development in the fetal conducting airway of the rhesus macaque.

Methods: We exposed animals to BPA during either the second (early term) or the third (late term) trimester. There were four treatment groups: *a*) sham control early term, *b*) sham control late term, *c*) BPA early term (BPA-early), and *d*) BPA late term (BPA-late). Because cellular maturation occurs nonuniformly in the lung, we defined mRNA and protein expression by airway level using microdissection.

Results: BPA exposure of the dam during late term significantly accelerated secretory cell maturation in the proximal airways of the fetus; both Clara cell secretory protein (CCSP) and MUC5AC/5B mRNA and protein expression increased.

Conclusions: BPA exposure during late gestation accelerates secretory cell maturation in the proximal conducting airways. We identified a critical window of fetal susceptibility for BPA effects on lung epithelial cell maturation in the third trimester. This is of environmental health importance because increases in airway mucins are hallmarks of a number of childhood lung diseases that may be affected by BPA exposure.

## Introduction

The respiratory health effects of bisphenol A (BPA) have been of recent interest (Kwak et al. 2009; Midoro-Horiuti et al. 2010; Nakajima et al. 2012; Roy et al. 2012). BPA is an organic chemical used in the production of polycarbonate plastics and epoxy resins. These plastics are found in food and drink packaging and are used as lacquers in food cans and bottle tops. BPA can migrate into food from these containers (Carwile et al. 2009; Rudel et al. 2011) and is also found in indoor air and dust (Inoue et al. 2006; Wilson et al. 2007). Over 90% of U.S. urine samples tested in the National Health and Nutrition Examination Survey (NHANES) have measurable levels of BPA (Calafat et al. 2008), indicating widespread and continual exposure. Exposure levels for adult humans are in the 0.3–22.3 ng/mL range for unconjugated BPA in serum (Padmanabhan et al. 2008; vom Saal et al. 2007), although a recent study found that consumption of canned soup resulted in short term 1,000-fold increases (Ohshima et al. 2007). Plasma BPA levels in pregnant women and in fetuses have a similar range (Schönfelder et al. 2002). In adult humans, BPA pharmacokinetics have been found to be similar to pharmacokinetics in mice and monkeys, with linear kinetics (Taylor et al. 2011) and fairly complete clearance; therefore, high serum levels in adult humans reflect continual exposures.

There is concern that current levels of exposure to BPA may adversely affect human development. In a companion study to our current research, BPA accelerated prenatal development of the rhesus monkey mammary gland, including increased mammary bud density and overall gland maturation, similar to what has been seen in rodent studies (Tharp et al. 2012; Vandenberg et al. 2007). In a mouse ovalbumin sensitization model, maternal exposure to BPA increased asthma hallmarks such as eosinophils in bronchoalveolar lavage fluid and airways hyperresponsiveness (AHR) in offspring (Midoro-Horiuti et al. 2010) although histology of the lung was not characterized. BPA is related to allergic sensitization in animal models and in humans (Chu et al. 2006; Midoro-Horiuti et al. 2010; Ohshima et al. 2007); however, lung effects have been little studied.

Many human lung diseases are characterized by abnormal epithelial cell secretions, particularly of mucus. Within the conducting airways, both mucins and Clara cell secretory protein (CCSP) have roles in airway disease (Ramsay et al. 2001; Voynow 2002), mature during pre- and postnatal development, and are among the most abundant secretory proteins in lung tissue. MUC5AC and MUC5B are the predominant secreted gel-forming mucins (Evans et al. 2009) with MUC5AC at as much as 300-fold lower levels than MUC5B during fetal lung development. CCSP is thought to have a protective role in the airways, regulating immune responses and attenuating oxidant stress (Plopper et al. 2005; Snyder et al. 2010). In general, mucin expression is more abundant in proximal airways and CCSP expression is more abundant in distal airways, corresponding to the differential abundance of mucous cells and Clara cells, respectively, in these airway regions. We selected CCSP and MUC5AC/B to study because these secretory proteins mature during the periods spanned by this fetal BPA exposure. Further, the rhesus monkey lung is an excellent model for human fetal lung development in that it recapitulates the cellular and anatomic composition, as well as the timing (Table 1), of human lung development (Plopper and Hyde 2008). In contrast, rodent models have airway secretory cells that are relatively immature at birth and do not contain mucous goblet cells throughout the tracheobronchial tree as the primary secretory cell type.

**Table 1:** Comparison of stages of fetal development in rhesus macaques and humans.^*a*^

Stage of pregnancy			Stage of lung development		
Trimester	GD		Lung stage	GD	
	Macaque	Human		Macaque	Human
First	≤55	≤90	Embryonic	21–55	
Second	55–110	90–180	Pseudoglandular	56–80	42–112
Canalicular			80–130	112–168
Third	110–165	180–270	Saccular	131–165	168–270
GD, gestation day. ^***a***^Based on data from Burri (1997), Plopper and Fannuchi (2004), and Tarantal and Gargosky (1995).					

Lung epithelial development occurs in a series of highly choreographed sequences of events that span the pre- and postnatal period (Plopper and Fannuchi 2004). Proximal conducting airway epithelial cells mature earlier than those in distal airways. Because prenatal lung development is site specific in the conducting airways and the late fetal period is one of dynamic change, we have incorporated site-specific methods into our analysis of conducting airway gene and protein expression. Exposure to toxicants during the prenatal period that disturb the normal course of development can result in disease later in life. The incidence of asthma is escalating in children, and there is a hypothesis that environmental factors may be related to the increasing incidence. Interestingly, as pointed out by Midoro-Horiuti et al. (2010), this rise in asthma prevalence (Vollmer et al. 1998) began 20 years after the widespread use of plastics began in the 1950s.

The effect of BPA on lung maturation in an animal model with cellular structure and airway architecture similar to humans, such as the rhesus monkey, has not been studied. The goal of the present study was to address three key issues: *a*) to define the normal pattern of expression of airway secretory proteins (CCSP, mucins) in the fetal rhesus monkey lung, *b*) to determine whether prenatal exposure to an environmentally relevant level of BPA changes the abundance of these key secretory proteins, and *c*) to determine whether there is a window of susceptibility for BPA effects on prenatal lung development.

## Methods

*Animals.* Adult female rhesus macaques (*Macaca mulatta*) were housed at the California National Primate Research Center as previously described (Hunt et al. 2012) [see Supplemental Material, p. 2 (http://dx.doi.org/10.1289/ehp.1206064)]. Animal protocols were approved by the Animal Care and Use Committee of University of California, Davis; all studies were conducted in accordance with the *Guide for the Care and Use of Laboratory Animals* (National Research Council Committee for the Update of the Guide for the Care and Use of Laboratory Animals 2011). Animals were treated humanely and with regard for alleviation of suffering.

**Figure 1:**
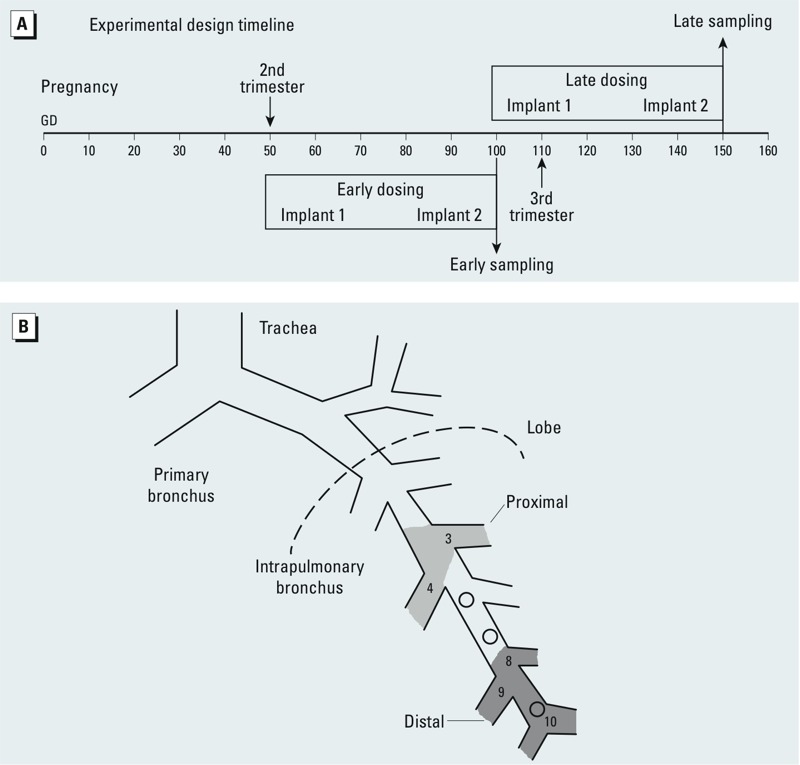
Timeline of BPA exposure (*A*) and the sampling scheme for microdissected airways in the lung (*B*). This study assessed two periods of BPA exposure in the fetus: an early exposure that ended on GD100 (second trimester) and a late exposure that ended on GD150 (third trimester). Age-matched sham-treated control animals were included, allowing analysis of normal fetal development as well as BPA effects. Exposure groups were *a*) sham control early term, *b*) BPA-early, *c*) sham control late term, and *d*) BPA-late. The lung diagram (*B*) illustrates the two airway sites sampled including intrapulmonary proximal bronchi generations 3–4 and distal airway generations 8 through respiratory bronchioles.

Only females (6–13 years of age) with a history of normal menstrual cycles were selected for this study. Animals were naturally mated. Pregnancy was detected by ultrasound examination and an estimated day of conception [gestation day (GD) 0] was assigned. At approximately GD40, the sex of all fetuses was determined and only those pregnancies with female fetuses continued in this study—the originating project for these samples was designed to study BPA effects on oogenesis. This study is part of a series of studies whose primary goal is to assess the effects of BPA on organogenesis in nonhuman primates using a dose that results in serum levels of BPA similar to those found in humans. Because of the expense of these studies, several laboratories shared tissues derived from the parent study. Tissues were obtained at GD100 and GD150 to study effects in the second and third trimester, respectively (Figure 1A).

*BPA dosing.* Deuterated BPA (dBPA; CDN Isotopes, Quebec, Canada) was used in this study because it can be clearly distinguished by isotope dilution liquid chromatography–mass spectrometry, thus eliminating concern about potential BPA contamination by materials used in the preparation, handling, or shipment of samples. dBPA in the dams was delivered in biocompatible silastic tubing implants placed subcutaneously via trocar in the scapular region so that the animals were continually exposed to BPA. Treatment days were at either mid-gestation (early dosing), GD50–GD100, or late gestation (late dosing), GD100–GD150 (see Figure 1A). Term is approximately GD165. We removed and replaced the implants with freshly prepared implants after about 25 days of treatment (halfway through the dosing period) to assure that BPA levels remained near the maximum release rate. Silastic tubing implants for each animal were prepared as previously described (Hunt et al. 2012). The calculated release rate of 1.056 mg/24 hr was based on test capsules loaded with tritiated BPA (^3^H-BPA) that were placed in saline solution for up to 40 days. The resulting serum levels for nonpregnant test animals that received implanted capsules for 2 weeks ranged from 2.2 to 3.3 ng/mL unconjuated dBPA, within the range (0.5–22.3 ng/mL) measured in humans (Padmanabhan et al. 2008). Age-matched control animals were treated with sham corn oil implants (*n* = 2 of each age). Additional lobes prepared similarly to those for quantitative real-time reverse transcriptase polymerase chain reaction (qRT-PCR) analysis in this study were available from age-matched sham-control animals given corn oil–treated fruit (*n* = 4–6 of each age). Control animals from the two studies did not vary from each other by treatment and so both control groups were combined and used together as controls for each age for qRT-PCR analysis (*n* = 7–8). The *in vivo* portion of the study was conducted with only two control animals assigned to each gestation age group (early and late) because of limited pregnant dam availability. We attempted to compensate for the small *n* by using both current and historical control data. However, the particular protocol for lung inflation with fixative and site-specific localization of airways used in the present study for histologic sample preparation differed significantly from historical control sample processing, making additional lobes from historic controls inappropriate for comparable histologic and immunohistochemical staining and subsequent morphometric analysis and resulting in *n* = 2 for each control group/age for these end points. BPA-treated samples were *n* = 6 for each age.

*Lung tissue processing.* All fetuses were removed by cesarean section at GD100 for the early-dosing group and GD150 for the late-dosing group. The lobes of the lung were subdivided and processed as described in Supplemental Material, p. 2 (http://dx.doi.org/10.1289/ehp.1206064). Because lung maturation occurs in a proximal to distal direction, we analyzed two groups of airway generations (Figure 1B) using qRT-PCR, high-resolution histopathology, and immunohistochemistry. The two groups were *a*) proximal airways (generations 3–4, intrapulmonary bronchi), and *b*) distal airways (airway generations 8–10, distal bronchioles).

*Immunohistochemistry and histochemistry.* Paraffin sections from two control animals and four treated animals per age (approximately 3–4 slides/animal) were immunostained for CCSP (1:2000; BioVendor, Asheville, NC). Controls included the substitution of primary antibody with phosphate buffered saline, which resulted in loss of specific staining [see Supplemental Material, Figure S1 (http://dx.doi.org/10.1289/ehp.1206064)]. Mucous cells were stained with Alcian Blue–Periodic Acid Schiff (AB/PAS) histologic stain (American MasterTech, Lodi, CA) following the manufacturer’s instructions (Caramori et al. 2009) (for details, see Supplemental Material, p. 2).

*Morphometric histopathology.* Because the amount of site-specific paraffin sections was limited in these fetal lungs, we quantified only the abundance of mucosubstance in the airway epithelium of proximal (intrapulmonary generations 1–3) conducting airways, determined using stereologic assessment of lung structure (Hsia et al. 2010). Paraffin sections (5-µm thick) from two to four animals per group per age (2–4 slides/animal) were stained for mucin using AB/PAS staining (Caramori et al. 2009). The volume fraction and mass of mucosubstance in the proximal epithelium, as well as epithelial thickness, were assessed in two controls and four BPA-treated animals per age [for details, see Supplemental Material, p. 3 (http://dx.doi.org/10.1289/ehp.1206064)].

*Gene expression. CCSP*, *Muc5AC*, and *Muc5B* gene expression was measured using qRT-PCR (*n* = 5–8) [for details, see Supplemental Material, pp. 3–4 (http://dx.doi.org/10.1289/ehp.1206064)].

*Statistics.* Fold change of gene expression in microdissected airways from 5–8 animals per time point was calculated using the comparative Ct method as described previously [Applied Biosystems; Life Technologies Corp., Carlsbad, CA (Livak and Schmittgen 2001)]. Results were reported as fold changes relative to proximal late control and graphed as mean ± SE. Statistical outliers were eliminated using the extreme studentized deviate method (Graphpad, La Jolla, CA). Undetected and samples observed below detection limit were treated as nondetects, and their values were imputed using the natural-log regression on order statistics method (Helsel 2005; Shumway et al. 2002) using ProUCL [U.S. Environmental Protection Agency (http://www.epa.gov/osp/hstl/tsc/software.htm)]. Multivariate analysis of variance (MANOVA) was applied against age, compartment, and exposure factors when appropriate. Pair-wise comparisons were performed individually using a one-way ANOVA followed by protected least significant difference (PLSD) post hoc analysis using StatView, version 5.0.1 (SAS Institute Inc., Cary, NC). *p*-Values of ≤ 0.05 were considered statistically significant. Morphometric analysis of proximal airway mucosubstance was assessed in control (*n* = 2) and BPA-exposed (*n* = 3–4) animals. Because of the small number of control animals (*n* < 3), there were not enough data to conduct rigorous statistical inferences between groups. Only descriptive statistics (arithmetic mean) are presented.

## Results

**Figure 2:**
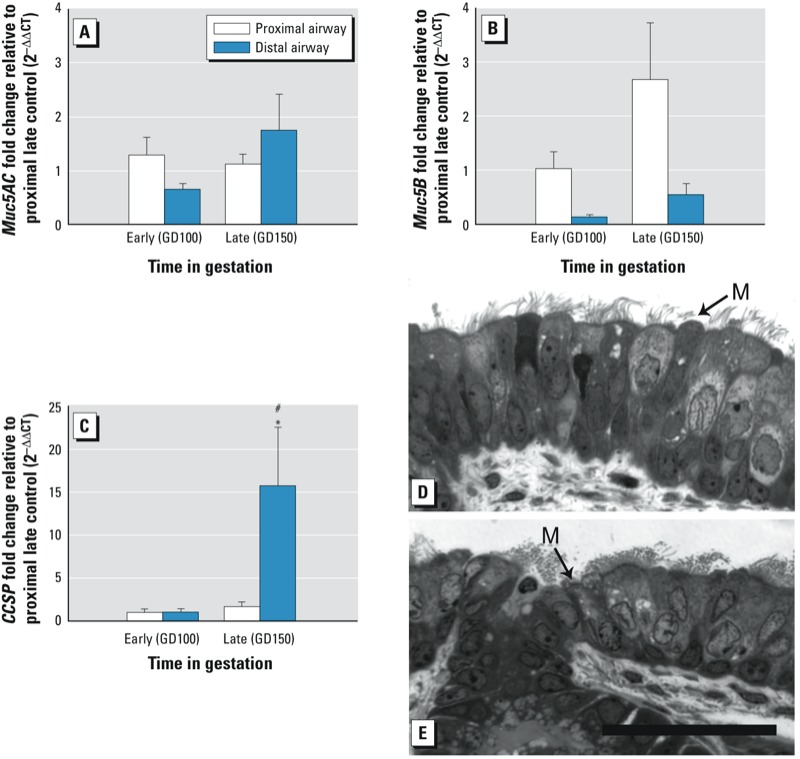
Expression of secretory products and epithelial morphology during normal prenatal development. Expression of secretory product mRNA in microdissected proximal and distal airways (*A–C*) as measured by qRT-PCR and reported as fold change compared with late control proximal airway. *Muc5* mRNA expression did not vary significantly but had slight, nonsignificant increases in *Muc5AC* (*A*) in distal airways (*p* = 0.07) and *Muc5B* (*B*) in proximal airways late in gestation compared with both early proximal (*p* = 0.4) and late distal compartments (*p* = 0.2). (*C*) *CCSP* gene expression was significantly increased late in gestation in distal airways in comparison with early distal (*p* = 0.001) and late proximal (*p* = 0.002) ages. (*D,E*) Representative high resolution histopathology of proximal airway epithelium in resin sections stained with methylene blue/azure II stain. Proximal airway epithelial cell morphology early in gestation (GD100) (*D*). Proximal airway epithelial cell morphology late in gestation (D150) (*E*). M, mucous cell. Data are presented as mean ± SE (one-way ANOVA and PLSD post hoc analysis). Bar = 50 µm. *p < 0.05 compared with same compartment, early age expression. #*p* < 0.05 compared with same age, proximal compartment expression. *n* = 5–8 for qRT-PCR.

*Normal expression of secretory products during prenatal development. Muc5AC* mRNA expression did not vary significantly by age or airway level (distal early vs. late *p* = 0.07), although a majority of samples (15 of 27) tested in distal airways at GD100 did not have detectable mRNA for this gene (Figure 2A). *Muc5B* mRNA did not differ significantly with age or compartment but was slightly more abundant in proximal airways versus distal airways late in gestation, at GD150 (Figure 2B). *CCSP* mRNA was significantly more abundant in distal bronchiolar airways (generations 8–10) (Figure 1B) at GD150 (*p* = 0.002) than in proximal airways or in airways earlier in gestation (*p* = 0.001) (Figure 2C). Maturation of the airway epithelium over the period of the present study was apparent on high-resolution resin sections. Glycogen, present as clear cytoplasmic inclusions in the tall pseudostratified epithelium, was more abundant at GD100 and the basement membrane was less marked at GD100 than at GD150 (compare Figure 2D with 2E). Mucous cells appeared more mature at GD150, with a protruding apex and a cytoplasm containing granules (Figure 2E).

**Figure 3:**
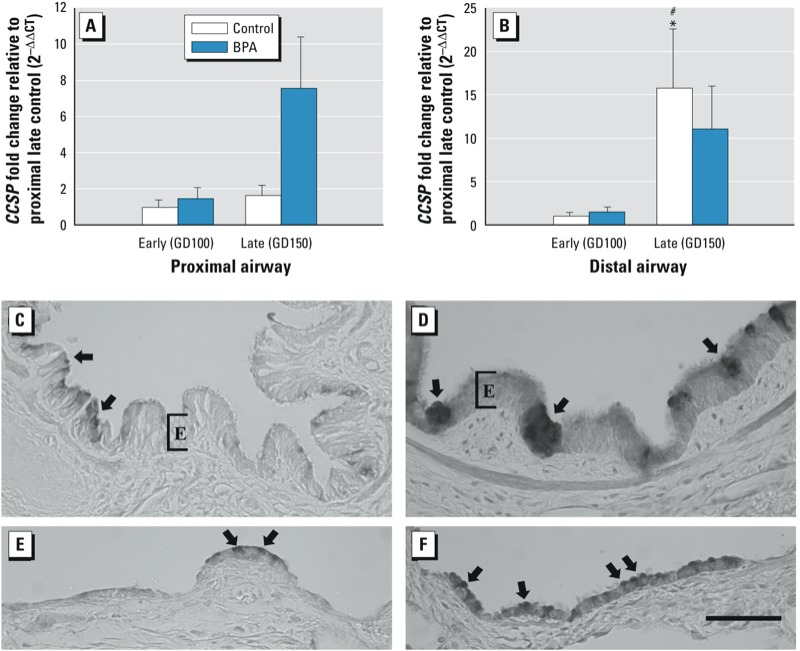
(*A,B*) Effect of BPA exposure on *CCSP* mRNA expression. *CCSP* mRNA expression in microdissected proximal (*A*) and distal airways (*B*) as measured by qRT-PCR and reported as fold change compared with late control proximal airway. Distal control (*B*) mRNA expression increased significantly with age (*p* = 0.001), and late distal control was also significantly greater than the age-matched proximal control (*A*) (*p* = 0.002). (*C–F*) Pattern of *CCSP* protein expression detected using immunohistochemistry on sections of proximal airways. Representative sections of late control (*n* = 2) (C) and late BPA-treated animals (*n* = 4) (*D*) show a substantial increase in *CCSP* protein expression in columnar epithelial cells with similar morphologic characteristics to mucous cells. *CCSP* protein in distal airways of late control (*E*) and late BPA exposed (*F*) was expressed in cells resembling Clara cells (arrows). E, epithelial cells. Data are represented as mean ± SE (one-way ANOVA and PLSD post hoc analysis). Bar = 50 µm. *p < 0.05 compared with same compartment, early age expression. #*p* < 0.05 compared with same age, proximal compartment expression. *n* = 5–7 for qRT-PCR values. Data are represented as mean ± SE (one-way ANOVA and PLSD post hoc analysis).

*Expression of secretory products after exposure to BPA. CCSP* mRNA was detected at all ages and in both proximal (Figure 3A) and distal (Figure 3B) airways. BPA exposure in late gestation resulted in an insignificant increase in *CCSP* mRNA expression in the proximal bronchi versus control (*p* = 0.2) (Figure 3A). Early gestation *CCSP* gene expression was unaffected by BPA in proximal or distal airways. In control animals, distal airways at DG150 contained significantly more *CCSP* mRNA expression than proximal (*p* = 0.002) or earlier GD100 (*p* = 0.001) airway levels (see Figure 3A, 3B). CCSP protein was localized to both tall pseudostratified epithelial cells of the large airways and simple cuboidal epithelium lacking cilia in the distal airways (Figure 3C–F). BPA exposure markedly increased the distribution and abundance of CCSP protein in the airway epithelium (Figure 3D and F).

**Figure 4:**
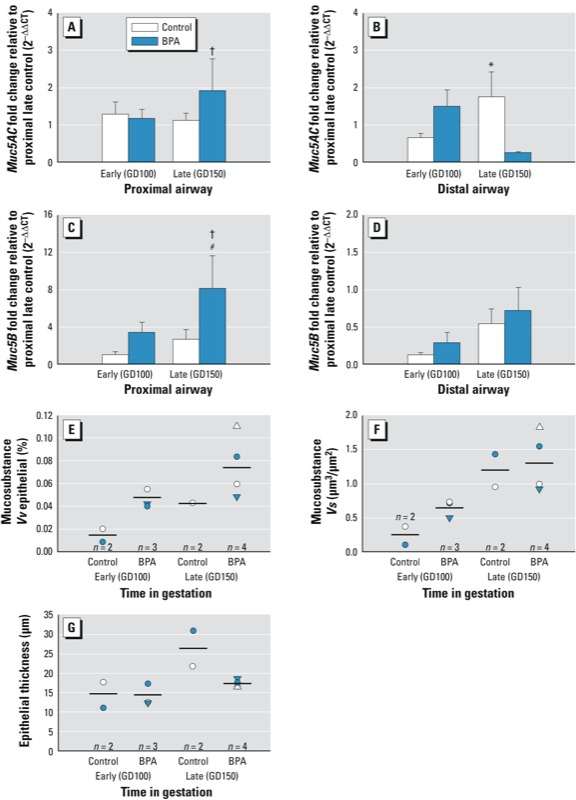
Effect of BPA exposure on mucin expression. *Muc5AC* (*A,B*) and *Muc5B* (*C,D*) gene expression was measured by qRT-PCR in microdissected proximal (*A,C*) and distal (*B,D*) airways. Gene expression changes are reported as fold change compared with late proximal control. Late proximal BPA (*A*) is significantly greater than matched distal (*B*) group (*p* = 0.02). Late distal control *Muc5AC* expression (*B*) was significantly greater than matched BPA (*p* = 0.02). Late proximal BPA *Muc5B* expression (*C*) was significantly greater than matched control (*p* = 0.005) and age- and treatment-matched distal group (*D*) (*p* = 0.003). Morphometric assessment of volume fraction, *Vv* (*E*), and volume per surface area (mass), *Vs* (*F*), of mucosubstance-positive proximal epithelial cells, and proximal epithelial thickness (µm) (*G*). Morphometric measurements of proximal airways are presented as individual data points (1/animal) with the bar representing the arithmetic mean (*n* = 2–4). BPA increases the volume fraction of mucosubstance in proximal epithelia in both early and late gestation (*E*) and the mass (*F*) of mucosubstance (*Vs*) in early gestation. Epithelial thickness decreased with BPA treatment in late gestation (*G*). Data are represented as mean ± SE (one-way ANOVA and PLSD post hoc analysis). *p < 0.05 compared with same compartment, BPA treated group. #*p* < 0.05 compared with same compartment, control group. †*p* < 0.05 compared with same age-treatment, distal compartment. *n* = 5–8 for qRT-PCR values.

*Muc5AC* mRNA levels were changed by airway level and BPA exposure (Figure 4A and B). Similar to effects on *CCSP* seen at GD150, late gestational exposure elicited no significant effect in proximal (*p* = 0.2) airways, but resulted in a significant decrease in distal (*p* = 0.02) airway mRNA expression. However, expression of *Muc5B* in the proximal bronchi was significantly increased in the BPA-late group by approximately 6-fold compared with GD150 control animals (*p* = 0.005) and distal BPA-GD150 airway expression (*p* = 0.003) (Figure 4C and D). Distal bronchiolar expression of *Muc5B* was not changed by age or BPA exposure.

**Figure 5:**
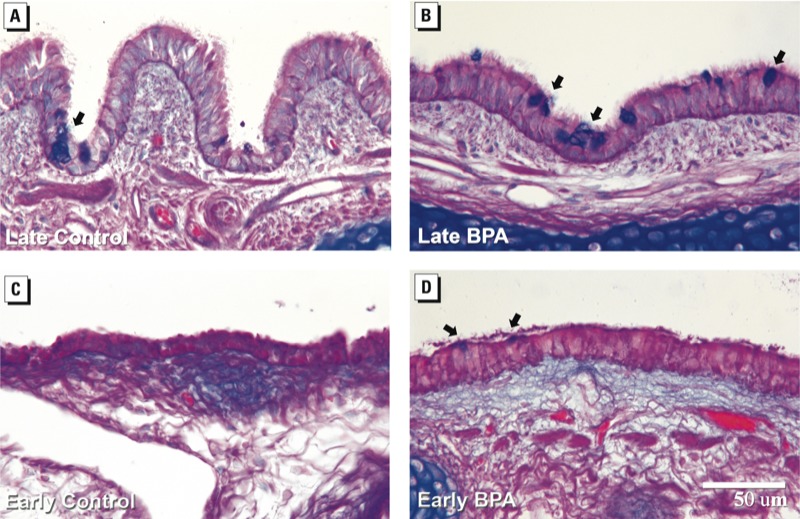
Effect of BPA exposure on proximal epithelial mucosubstance expression. Representative sections of airway epithelial mucosubstances, as detected using AB/PAS staining in proximal airways of late control (*A*), late BPA (*B*), early control (*C*), and early BPA (*D*) exposed groups. Mucosubstance was ­localized to cells resembling goblet cells (arrows). Bar = 50 µm.

Mucins detected using AB/PAS histologic staining indicated mucosubstance-positive cells in proximal airway epithelia. Morphometric assessment of volume fraction, *Vv* (Figure 4E), and volume per surface area or mass, *Vs* (Figure 4F), of mucosubstance-positive cells and proximal epithelial thickness (*t*, in micrometers) (Figure 4G), showed that all three parameters increased with age. BPA exposure enhanced this trend by increasing volume fraction in both early and late gestation (Figure 4E) but only increased mass in the BPA-early group (Figure 4F). BPA late in gestation reduced epithelial thickness compared with matched controls to levels just above the early groups. Figure 5 shows increased incidence of mucosubstance-positive cells in the proximal airways of BPA-exposed animals compared with controls during both late (compare Figure 5A with Figure 5B) and early (compare Figure 5C with Figure 5D) gestation.

## Discussion

Our data indicate that exposure to environmentally relevant levels of BPA during fetal lung development can alter expression of secretory genes (*Muc5B, CCSP*) and proteins (MUC5B, CCSP) in the conducting airways. Further, we found that this increase was most pronounced in the proximal conducting airways, bronchi. BPA exposure later in gestation (roughly spanning the third trimester) has a greater effect on epithelial secretory maturation than an earlier exposure. Thus we have identified a critical window of timing in development for BPA alteration of the normal lung. It is likely that this critical window of time will also apply to human exposures in the third trimester because the timing of cellular development, as well as conducting airway architecture/cellular composition, in the rhesus monkey lung closely recapitulates that in humans (Table 1) (Burri 1997; Plopper and Hyde 2008; Tarantal and Gargosky 1995). Our results also underscore the importance and feasibility of using site-specific methods to study fetal development in the rhesus monkey: Cell maturation in the conducting airways occurs in a proximal to distal direction, comparing like sites is important because of the large gradient in differentiation between different airway generations.

There is a dichotomy in BPA’s effects on conducting airway mucins: MUC5B is affected by exposure, and MUC5AC expression is not. Mucins are critical for maintenance of normal lung homeostasis. They contribute to the liquid lining layer of the airways and assist with the removal of foreign substances and the regulation of inflammation. Overly abundant secretion and storage of mucus can cause airway obstruction as found in a number of lung diseases, including asthma and bronchitis. BPA exposure increases both mucous cell abundance and *Muc5B* gene expression. BPA exposure increases the percentage of mucous cells (*Vv*; Figure 4E), but the mass of mucous cells (*Vs*; Figure 4F) is increased only in the early exposure group. This is possibly due to decreased epithelial thickness (*t*; Figure 4G) in the BPA late exposure group, when the mucous cells make up a higher percentage of a thinner epithelium. The effects of BPA exposure on *Muc5B* may be due to the binding of the parent molecule or its metabolites to estrogen receptors (Okuda et al. 2011). Estrogen (17β-estradiol) is known to induce *Muc5B* expression in airway epithelial cells (Choi et al. 2009) via estrogen receptor-α. BPA interacts with both nuclear estrogen receptors-α and -β, which regulate transcription as well as cell membrane–bound estrogen receptors (vom Saal et al. 2007). MUC5B is found both in airway submucosal glands and in surface epithelial goblet cells (Finkbeiner et al. 2011). MUC5AC is more predominant in the surface goblet cells (Finkbeiner et al. 2011). We were not able to analyze the effects of BPA on glandular development; we had too little sample to define this histologically and the airway microdissection method we used for qRT-PCR combines both airway glands and the surface epithelium in the same sample. Future studies could correlate glandular versus airway epithelial expression of MUC5B using laser capture microdissection as has been done in the study of salivary glands in humans (Kouznetsova et al. 2010). Our data shows that BPA exposure increases the expression of both the gene and the protein for the two most abundant secretory proteins, MUC5B and CCSP, in the airways. Increased expression is apparent with more cells containing the protein, increased abundance of the protein per cell, and increased gene expression on an airway basis.

The biologic relevance of the increase in CCSP in the proximal airways is unknown. There is little data showing the effects of increased CCSP. However, decreased secretion of CCSP has been found in the lavage fluid of patients with asthma (Van Vyve et al. 1995), and polymorphisms in this gene that confer low serum levels of CCSP correlate with an increased risk of asthma in children with allergic rhinitis (Ku et al. 2011). In general, CCSP is considered a beneficial protein, so much so that recombinant human CCSP has been considered as a therapy in infants with respiratory distress (Abdel-Latif and Osborn 2011). CCSP has not been reported to be responsive to estrogens in the lung but can be increased by exposure to interferon-γ (Ramsay et al. 2003) and tumor necrosis factor-α (Cowan et al. 2000). Why CCSP is up-regulated by BPA in the epithelium of the large airways of fetal rhesus monkeys will require further investigation.

It is unknown whether increased expression of MUC5B and CCSP is an aberrant process that could persist and lead to pathology or disease later in life or whether this is actually a neutral or even beneficial process. This is a limitation of the present study, which does not contain a follow-up period succeeding BPA exposure to determine whether these changes persist. What makes the current findings worrisome, however, are previous studies that demonstrate fetal BPA exposure increases allergic sensitization and asthma hallmarks in mouse models (Midoro-Horiuti et al. 2010; Nakajima et al. 2012). In the mouse, when BPA exposure spanned the period from before implantation to weaning, BPA exposure accelerated airways AHR to allergen challenge and increased eosinophils in the lavage fluid in the offspring of BPA-exposed dams (Midoro-Horiuti et al. 2010). A follow-up study also found AHR after a shorter BPA exposure period that included only the prenatal period, from pre-implantation to birth (Nakajima et al. 2012). Our exposure paradigm is a still shorter period, spanning most of a trimester late in gestation; nevertheless, it shows significant effects on the lung. BPA also affects the immune system, leading to speculation that BPA may be involved in the development of asthma and allergy (Kwak et al. 2009). BPA has been shown to increase interleukin-4 production in primed CD4^+^ T cells (helper T cells) and also increases antigen-specific immunoglobulin E in primed mice, potentially enhancing allergic responses (Lee et al. 2003). BPA exposure has also been found to slightly alter innate immunity in mice exposed to influenza (Roy et al. 2012). If mucous cell abundance is increased by BPA, and AHR/allergy also is increased, this could synergize and increase airway obstruction, making asthma more severe. Future studies of the effects of BPA on lung cellular development and asthma are needed and should focus on exposures that encompass this late fetal period and also include prolonged follow-up to determine long-term effects of early-life exposure to BPA.

The present study found significant effects in the fetus when the dam was exposed to BPA. Significant effects of chemical exposure during prenatal development occur for many reasons: a critical window of susceptibility, an enhanced delivered dose to the fetus due to differences in fetal-maternal detoxification/metabolism, formation of unique metabolites, or selective uptake by compartments unique to the fetus including amniotic fluid or placenta. It is unknown whether all or some of these factors contribute to BPA’s fetal effects on the lung in this model. Certainly the chemical composition of the amniotic fluid should be considered because this fluid bathes the epithelium lining the lung, which is affected by prenatal BPA exposure. The lung contains substantial xenobiotic metabolizing enzymes that can contribute to the local burden of metabolites and have transient expression during lung development. This is of interest because some BPA metabolites have more estrogenic activity than the parent molecule (Nakamura et al. 2011). Cytochrome P450 monooxygenases mature late in development and localize to the epithelial lining layer of the respiratory tract, the very area shown here to be affected by fetal BPA exposure. BPA is metabolized by cytochrome P450s and is detoxified through glucoronidation and sulfation. The balance of activation and detoxification is likely an important determinant of BPA effects, and this includes both maternal and fetal capabilities. Studies are needed to define the relative role of these enzymes and their influence on pharmacokinetics in the prenatal period, particularly during the third trimester in primates.

It is important to acknowledge the limitations of this study. The sample is small for the histologic end points, and, although the sample is larger for the significant gene expression data, replication and extension of the study would provide more confidence in the study results. There is a lack of an exposure group that is followed into the postnatal period, which would allow for assessment of persistence of effect as well as study of pulmonary function and lung compliance. Finally, because the original study was designed to look at effects on oogenesis, the monkey fetuses in this study are all female. Future studies should include both sexes because asthma is more prevalent in prepubertal males (Vink et al. 2010).

## Conclusions

BPA exposure during late gestation accelerates secretory cell maturation in the proximal conducting airways. We have identified a critical window of fetal susceptibility for BPA effects on lung epithelial cell maturation in the third trimester of a highly relevant model, the rhesus monkey. This is of environmental health importance because increases in airway mucins are hallmarks of a number of childhood lung diseases that may be affected by BPA exposure.

## Supplemental Material

(1.5 MB) PDF
