# Supplementary material for: Fetal Exposure of Rhesus Macaques to Bisphenol A Alters Cellular Development of the Conducting Airway by Changing Epithelial Secretory Product Expression
Source: Environ Health Perspect. 2013 Jun 11;121(8):912–8. doi: 10.1289/ehp.1206064 (PMC3734491; doi:10.1289/ehp.1206064)
Supplement: (1.5 MB) PDF [file ehp.1206064.s001.pdf]

Environmental Health Perspectives doi:10.1289/ehp.1206064

SUPPLEMENTAL MATERIAL

Fetal Exposure of Rhesus Macaques to Bisphenol A Alters Cellular Development of the Conducting Airway by Changing Epithelial Secretory Product Expression

Laura S. Van Winkle, Shannon R. Murphy, Miriam V. Boetticher and Catherine A. VandeVoort

Table of Contents

METHODS

Animals Page 2

Lung Tissue Processing Page 2

Immunohistochemistry and histochemistry Page 2

Morphometric histopathology Page 3

Gene Expression Page 3

Supplemental Figure S1 Page 5

REFERENCES Page 6

METHODS

Animals: Animals were caged individually with a 0600–1800 hours light cycle with temperature maintained at 25–27°C. Animals were housed in stainless steel cages, fed a diet of Purina Monkey Chow, given water *ad libitum* via "lixit" device and provided seasonal produce, seeds and cereal as enrichment.

Lung Tissue Processing: The right cranial lobe was cannulated at the lobar bronchus and instilled with 1% paraformaldehyde at 25cm constant pressure for 1h. Tissue was fixed overnight (approximately 18 hrs) and the axial path airways microdissected and the generations noted. Necropsies occurred on different days and care was taken to fix all lung tissue for the same length of time to allow comparison staining intensity. For high resolution histopathology, the right caudal lung lobe was cannulated and inflated at 25cm constant pressure with Karnovsky's fixative (0.9% glutaraldehyde/0.7% paraformaldehyde in cacodylate buffer, adjusted to pH 7.4, 330 mOsm) for 1h. Fixed lungs were sliced into cross sections and embedded in Araldite 502 resin. Blocks were sectioned at 1 micron and sections stained with methylene blue azure II (Van Winkle et al. 2004). The left cranial lung lobe was cannulated, inflated to capacity with RNALater and stored at -20°C until used for airway microdissection and qRT-PCR as described in (Baker et al. 2004).

Immunohistochemistry and histochemistry: CCSP immunostaining- we used the avidin-biotin peroxidase method to detect primary antibody binding to the protein with a Vector ABC Kit following manufacturer's instructions. Nickel enhanced DAB (3,3'-diaminobenzidine; Sigma Chemical, St Louis MO) chromagen was used. A dilution series was used to determine optimal antibody concentration.

Morphometric histopathology: Whole slides were scanned at x40 on an Olympus VS110 virtual microscopy system scanner (Olympus, Tokyo, Japan). Scanned images were evaluated at x40 resolution for volume fraction and at x10 resolution for surface per volume. Standard morphometric approaches were used (Hsia 2010).The volume fraction of mucosubstance-positive cells was estimated using point (P) and intercept (I) counting of bronchial epithelium, utilizing vertical uniform random sine weighted probes and Visiopharm Integrator System software (Visiopharm, Hoersholm, Denmark). Volume fraction was calculated as: $V_{v\,(\mathrm{epi})} = P_p = \Sigma P_n/\Sigma P_t$ where P_p is the point fraction of P_n, the number of test points hitting the positive epithelial cells, divided by P_t, the total points hitting the reference space (epithelium). To calculate the surface area of epithelial basement membrane per reference volume (S_v) by point and intercept counting we used the formula: $S_{v\,\mathrm{bl}\,(\mathrm{epi})} = (2\,\Sigma I_{bl})/((l/p)\,\Sigma P_{t\,(\mathrm{epi})})$ where I_{bl} is the number of intersections with the reference space (epithelial basal lamina), P_t is the number of points hitting the epithelium and l/p is the length of cycloid test line per test point on the epithelium. Mucosubstance volume within the epithelial compartment per surface area of basal lamina ($\mu m^3/\mu m^2$) was calculated using the formula: $V_{s,\mathrm{bl}} = V_{v\,(\mathrm{epi})} / S_{v\,\mathrm{bl}\,(\mathrm{epi})}$. To estimate the mean arithmetic mean thickness of airway epithelium, we used the formula $\bar{\tau}_{epi} = V_{(\mathrm{epi})}/S_{(\mathrm{epi})}$, where $V_{(\mathrm{epi})}$ is the point count estimated volume of the epithelium and $S_{(\mathrm{epi})}$ is the point and intercept estimated surface area of the epithelial basal lamina per volume of epithelium.

Gene Expression: We isolated RNA using the Qiagen RNeasy Plus Mini Kit (Qiagen, Valencia, CA) and yield was assessed with a Nanodrop fluorospectrophotometer (Nanodrop, Wilmington, DE). Average yield: 0.2ug. An Applied BioSystems Step One Plus PCR System (Applied Biosystems, Inc., Foster City, CA) was used to generate cDNA and perform qRT-PCR, using Applied BioSystems Taqman® reagents, probes and primers. Purified RNA samples were stored

at -80°C until processed. cDNA (50ng) was made with the Taqman® cDNA RT-Transcription Kit (Applied Biosystems, Inc., Foster City, CA) and stored at -20°C. Reaction protocol: 25°C for 10 minutes, 48°C for 30 minutes, 95°C for 5 minutes then 4°C. All qRT-PCR samples were run in triplicate with RPL13A, a custom primer and probe assay, as the internal reference gene as this gene is recommended as a reference gene for rhesus monkey tissues. The sequences for RPL13A were: 5' primer CACGACGTTGGCTGGAAGT, 3'primer TCTTTCCTCTTCTCCTCCAAGGT and probe CCAGGCAGTGACAGC (Ahn et al. 2008). The CCSP, Muc5B and Muc5AC reactions used inventoried Taqman® probe/primer assays (CAT# 4331182: Hs00171092_m1 and Hs00861588_m1; CAT#4331348: Hs00873651_mH, respectively). The reaction protocol was as follows: 1 cycle at 95°C for 20 seconds, 50 cycles at 95°C for 3 seconds then 60°C for 30 seconds.

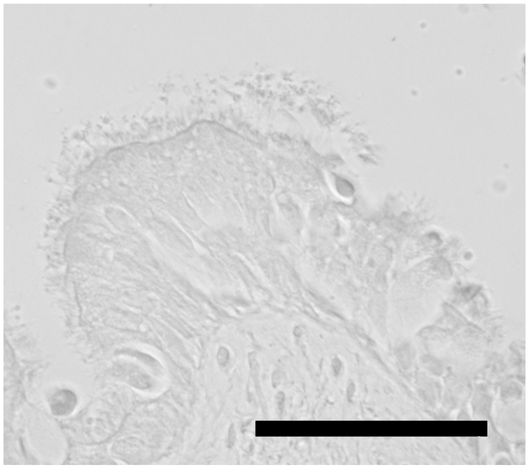

Supplemental Figure S1: When PBS was substituted for primary antibody, positive epithelial staining was eliminated, demonstrating lack of non-specific binding in tissue sections. Late control proximal airway from a CCSP immunohistochemical assay shows no staining in the absence of primary antibody. Magnification bar = 50 um.

REFERENCES

Ahn K, Huh JW, Park SJ, Kim DS, Ha HS, Kim YJ, et al. 2008. Selection of internal reference genes for sybr green qrt-pcr studies of rhesus monkey (macaca mulatta) tissues. BMC Mol Biol 9:78.

Baker GL, Shultz MA, Fanucchi MV, Morin DM, Buckpitt AR, Plopper CG. 2004. Assessing gene expression in lung subcompartments utilizing in situ rna preservation. Toxicol Sci 77:135-141.

Hsia CCW, D. M. Hyde, M.Ochs, and E.R. Weibel. 2010. An official research policy statement of the american thoracic society/european respiratory society: Standards for quantitative assessment of lung structure. Am J Respir Crit Care Med 181:394-418.

Van Winkle LS, Fanucchi MV, Miller LA, Baker GL, Gershwin LJ, Schelegle ES, et al. 2004. Epithelial cell distribution and abundance in rhesus monkey airways during postnatal lung growth and development. J Appl Physiol 97:2355-2363.
